# Evaluation of Urinary Tract Infection following Corticosteroid Therapy in Patients with Multiple Sclerosis Exacerbation

**DOI:** 10.1155/2021/6616763

**Published:** 2021-01-31

**Authors:** Aliyeh Bazi, Seyed Mohammad Baghbanian, Monireh Ghazaeian, Sahar Fallah, Narjes Hendoiee

**Affiliations:** ^1^Department of Clinical Pharmacy, Faculty of Pharmacy, Mazandaran University of Medical Sciences, Sari, Iran; ^2^Department of Neurology, Faculty of Medicine, Mazandaran University of Medical Sciences, Sari, Iran; ^3^Department of Biostatistic, Ibne Sina Medical and Educational Center, Mazandaran University of Medical Sciences, Sari, Iran

## Abstract

The first treatment for multiple sclerosis exacerbation is usually short-term intravenous methylprednisolone (IVMP), with or without a regimen of oral prednisone taper (OPT). This study aims to evaluate the effects of IVMP and OPT in comparison with IVMP alone in raising the risk of urinary tract infection (UTI) and posttreatment improvement of urinary tract symptoms in patients with relapsing-remitting multiple sclerosis. This double-blind randomized clinical trial was conducted on 56 people with multiple sclerosis relapse who had undergone methylprednisolone for 5 days. Patients were randomly split into two groups: oral prednisolone and placebo (tapering for 20 days). Demographic data, duration of multiple sclerosis, urinary tract symptoms, the Expanded Disability Status Scale (EDSS) score, and urine data were analyzed. The incidence of UTI in the intervention and control groups did not differ significantly (*p*=560). However, the improvement of urinary tract symptoms in the intervention group was significantly more favorable than in the control group (*p* ≤ 0.001). Furthermore, administering OPT after IVMP did not increase the risk of UTI occurrence in patients with multiple sclerosis exacerbation. The urine analysis results did not show any differences at baseline and after the corticosteroid tapering regimen. Due to the risk of infection by corticosteroids, it is no longer necessary to do further urinary screening in this group of patients.

## 1. Introduction

Multiple sclerosis (MS) is a chronic inflammatory disease of the central nervous system that is characterized by the presence of demyelinating plaques in the brain and spinal cord [1]. It is one of the most prevalent neurological diseases in young adults, particularly women [2]. Studies show that about two million people worldwide are affected by this illness [3].

MS often appears in its relapse-remitting form [4]. Exacerbation of MS is described as the beginning of a new neurological complication or worsening of earlier ones, which last for 24 hours or more if they are not caused by fever or infection [5]. On the other hand, pseudorelapse lasts less than 24 hours and is not associated with demyelinating changes. In addition, pseudorelapse is only an old symptom that is exacerbated by systemic stresses such as temperature changes, fever, and infection [6, 7].

Urinary tract infection (UTI) aggravates neurological symptoms in MS patients. It is both one of the main factors leading to pseudorelapse and one of the main causes of mortality and morbidity in MS patients [8–13]. UTI refers to the occurrence of fever, chills, dysuria, frequent urination, suprapubic pain, and pathologic urinalysis or positive culture [10]. Factors such as sphincter dysfunction, female gender, incomplete bladder depletion, and constant or occasional use of a urinary catheter augment the risk of this infection in people with MS [13–15]. Also, the longer the MS duration and the higher the Expanded Disability Status Scale (EDSS) score, the greater the risk of UTI in these patients [14].

Today, the first-line treatment of MS exacerbations is prescribing high doses of corticosteroids [16]. One of the serious adverse effects of using corticosteroids is an increased risk of infection or masking its symptoms. Due to immunosuppression effects, this class of drugs impairs cellular immunity and makes a person susceptible to a variety of microorganisms. Such risks are associated with the dose and duration of treatment with corticosteroids [17, 18].

This is a placebo-controlled, double-blind clinical trial. It aims at evaluating the effects of oral prednisolone tapering (OPT) after intravenous methylprednisolone pulse (IVMP) therapy on the risk of UTI in patients with multiple sclerosis relapse.

## 2. Materials and Methods

### 2.1. Study Design

This is a randomized prospective double-blind study with a placebo control that was conducted between October 2019 and February 2020 on patients with MS exacerbation admitted to Bu Ali Hospital, Sari. It was registered in the Iranian Registry of Clinical Trials (IRCT20120314009297N6) and was approved by the Ethics Committee of Mazandaran University of Medical Sciences (IR.Mazums.Rec.1398.5163).

MS exacerbation was treated with 1 g of IVMP for 3–5 days with or without an OPT, starting at 50 mg a day and tapered over 14–20 days. On the basis of the inclusion and exclusion criteria, participants were randomly assigned to one of two treatments: oral prednisolone (Abureihan CO, Iran, Tehran) group and placebo group. In the intervention group, OPT was administered at 50 mg taken once daily after breakfast and was tapered over 20 days. All the conditions of the intervention group were maintained for the control group, except that they received a placebo as an alternative to prednisolone tablets. The study was done in accordance with the declaration of Helsinki. After knowing the purpose of the study, all the patients provided their written informed consent prior to recruitment.

### 2.2. Inclusion and Exclusion Criteria

Inclusion criteria: Patients above 18 years old with moderate MS exacerbation who were treated with 1 g of IVMP for 5 days.

Exclusion criteria: UTI during multiple sclerosis relapse, urinary catheterization, neurogenic bladder, pregnancy and breastfeeding, underlying kidney disease, prednisolone allergy, and antibiotic use in the past two weeks.

### 2.3. Intervention

The recruited patients underwent neurological examinations. As part of the bowel/bladder Functional System (FS) score, they were asked about the presence of urinary tract symptoms including urgency, frequency, hesitancy, urge incontinence, and urinary retention. Neurologic disability was assessed using the EDSS. Next, we recorded patients' characteristics including gender, age, marital status, and duration of MS, disease-modifying drugs (DMDs), and clinical urinary tract symptoms (such as urinary retention, dysuria, urinary incontinence, and frequency/urgency). Urinalysis was completed within 1 h after receiving samples; if the results were negative for UTI, the neurologist would prescribe the coded packaging.

In this study, UTI was defined as the persistence of bacteriuria (bacteria counts were reported many) and pyuria (WBC more than 5/hpf) or positive nitrite in urine analysis.

We used a manual urinalysis system for dipstick and microscopic testing. The parameters assessed were nitrite by the test strips (Bahar Afshan CO, Tehran, Iran) and white blood cells (WBCs) and bacteria by microscopy. WBC and bacteria were counted per high-power field (hpf, 400× magnification), but bacteria counts were reported quantitatively as few (less than 20 bacteria observed in each field), moderate (the middle ground between these two terms), and many (employed when the number of bacteria in each microscope field was such that no space could be observed). Microscopic analysis was regarded positive for pyuria when the number of WBC was >5/hpf.

After a 20-day follow-up session on the 25th day of corticosteroid administration, urinalysis was repeated and patients were examined by the same neurologist and interviewer to evaluate the presence of urinary tract symptoms and EDSS.

### 2.4. Outcomes

The primary outcome was the occurrence of UTI. Secondary endpoints were measured based on the EDSS score and the symptomatology of urinary. These endpoints were assessed on the 20th day after the onset of the study.

### 2.5. Sample Size, Randomization, and Blinding

A sample size of 56 (28 in each group) was calculated based on the standard deviation (SD) of 1.3 [19], the statistical power of 80%, and alpha of 0.05. The patients were randomized through a simple computerized randomization program to receive either OPT or placebo. The patients, pharmacist, nurses, a neurologist, and the statistician were blind to group allocation. The medicine was used for both groups (intervention and placebo) in the same package with the coded label.

### 2.6. Statistical Analysis

Statistical analysis was performed using SPSS 24.0. (IBM Corp., Armonk, NY). Frequencies were then calculated for each variable. All interval variables were tested for normality of distribution using Kolmogorov–Smirnov test. The data is presented using means and standard deviation. Values sampled from normal distributions were appropriately compared using a Student's *t*-test. Values without normal distributions were compared by nonparametric tests (Mann–Whitney-U test). Qualitative variables were expressed in percentage and were compared using the Chi-square test and Fisher's exact test. Only measures with *p* < 0.05 were considered statistically significant.

## 3. Results

**Figure 1:**
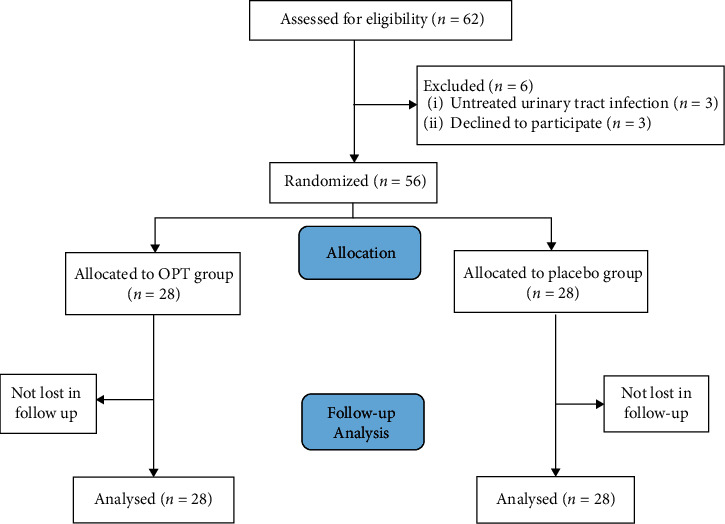
Randomization, treatment, and follow-up procedures.

A total of 62 patients with MS exacerbation were screened for eligibility, of whom 56 patients who met the inclusion criteria and consented to participate in the study, were randomized into two groups of 28 individuals (Figure 1). Six patients were excluded because three of them did not provide their informed consent and three others could not meet the inclusion criteria. All the patients had relapsing-remitting MS. The baseline characteristics are shown in Table 1.

The two groups together comprised 21 (36.8٪) males and 35 (61.4٪) females. In the intervention group, 19 were females and 9 were males, while the control group included 16 females and 12 males. Gender distribution was not significantly different in the OPT and placebo groups (*p*=0.687). The median age of participants was 30.57 ± 5 in the intervention group and 32.76 ± 7.09 in the control group, which was not significantly different (*p*=0.207; Table 1).

The two groups showed no significant difference in terms of EDSS score before (*p*=0.834) and after (*p*=0.677) the intervention (Table 2). Nevertheless, EDSS score decreased significantly in the OPT group on the 25th day of the study (*p* ≤ 0.001) (Table 1).

The frequency of urinary tract symptoms was greater in the OPT group than in the control group (42.9% versus 28.6%), and significant differences occurred between the two groups at baseline (*p*=0.033) (Table 1). However, the rate of urinary symptoms improvement was significantly higher in the OPT groups than in the control group (*p* ≤ 0.001) (Table 2).

No UTI was observed in the intervention and control groups at baseline. At the end of the study, respectively, one and three cases of UTI were detected in the intervention and control groups, but this difference was not statistically significant (*p*=0.560) (Table 2**).**

## 4. Discussion

Corticosteroids have been utilized for around 60 years to treat MS exacerbation [19]. There are concerns about the occurrence of pyelonephritis and urosepsis in people with MS relapse and untreated UTI who receive corticosteroids [20]. Although some studies have demonstrated the side effects of methylprednisolone in patients with MS exacerbation, to the best of our knowledge, this study is the first clinical trial to evaluate the relationship between OPT after IV methylprednisolone and increased risk of UTI in this group of patients.

Comparing the risk factors of UTI, such as the female gender, duration of MS, and EDSS score at the beginning of the study, showed no differences between the two groups. Additionally, the two groups did not differ in dose and duration of IVMP. Although marital status is a predisposing factor for UTI [21], the findings of our study revealed no correlation between this variable and UTI.

Despite common urinary symptoms reported by some patients at the beginning of the study, the urinary analysis did not confirm UTI diagnosis. It could be inferred that UTI diagnosis exclusively based on urinary signs in patients with MS can be unreliable, which is in line with another previous study [22]. Moreover, the rate of improvement of urinary symptoms in the intervention group was greater than that in the control group, which means that using OPT for a short time after MS exacerbation is not an additional risk factor for urinary infection. Nonconventional MRI (magnetic resonance imaging) studies have shown that the entire central nervous system is inflamed at different rates during MS attacks [23]. However, one should not rule out the possibility of worsening previous symptoms during a new attack. Therefore, improvement in urinary symptoms with corticosteroids may be a sign of reduced overall inflammation in MS attacks.

The results of other studies suggest that the suppression of the immune system with corticosteroids has a lower effect on the infectivity of uropathogens (mostly *Escherichia coli*) than on respiratory microorganisms (e.g., *Streptococcus pneumonia* and *Haemophilus influenza*) [24, 25]. Although the use of corticosteroids is associated with new or recurrent infections [17, 26, 27], our results of urine analysis exhibited that corticosteroid tapering to treat MS exacerbation does not increase the risk of UTI. While corticosteroids alone do not induce UTI, excluding pseudorelapse is vital to preventing any complications before IVMP.

Our study has some limitations. In this regard, the small sample size and lack of long-term follow-up to assess the effects of corticosteroid tapering regimen on clinical symptoms recurrence or any side effects could have affected our interpretation of the results.

## 5. Conclusion

Oral corticosteroid tapering after IVMP to manage MS exacerbation is not associated with an increased risk of UTI. Further urinary examinations are not necessary after tapering therapy due to their low risk of infection recurrence.

## Figures and Tables

**Table 1:** Baseline characteristics of the study population.

Parameter	OPT group	Control group	*P* value
Age (mean ± SD)	30.57 ± 5	32.76 ± 7.09	0.207
Gender ratio (female/male)	19/9	16/12	0.687
Disease duration	2.60 ± 1.96	4.67 ± 4.48	0.174
DMDs before relapse *n* (%)			
Glatiramer acetate	1	5	.084
Interferon beta 1b	0	1	1.000
Interferon beta 1a	8	6	0.537
Dimethyl fumarate	1	2	0.553
Fingolimod	2	1	0.553
No previous use of DMDs	16	13	0.422
EDSS (mean ± SD)	3.21 ± 1.40	3.28 ± 1.13	0.834
Marital status (married)	53.6%	71.4%	0.626
Urinary tract symptoms, *n* (%)	12 (42.9%)	8 (28.6%)	0.033

MS: multiple sclerosis; RRMS: relapsing-remitting multiple sclerosis; OPT: oral prednisone taper; SD: standard deviation; DMDs: Disease-Modifying Drugs; EDSS: Expanded Disability Status Scale.

**Table 2:** Pre- and postintervention values for the number of UTIs, EDSS score, and urinary tract symptoms in the two study groups.

Parameter	OPT group			Control group			*P* ^b^
	Preintervention	Postintervention	*P* ^a^	Preintervention	Postintervention	*P* ^a^	
Number of UTIs	0	1	—	0	3	—	0. 560

EDSS score (mean ± SD)	3.21 ± 1.40	1.53 ± 1.06	≤.001	3.28 ± 1.13	1.67 ± 1.46	≤.001	0.677

Urinary tract symptoms (%)	[12] 42.9%	[3] 10.7%	0.012	[8] 28.6%	[8] 28.6%	1.000	≤.001

UTI: urinary tract infections; EDSS: Expanded Disability Status Scale; OPT: oral prednisone taper; SD: standard deviation. P^a^: *P* values between pre- and postintervention in each group; P^b^: *P* values after intervention in the two groups.

## Data Availability

The data of the study are available from the corresponding author upon rational request.
